# Ionic-electronic halide perovskite memdiodes enabling neuromorphic computing with a second-order complexity

**DOI:** 10.1126/sciadv.ade0072

**Published:** 2022-12-23

**Authors:** Rohit Abraham John, Alessandro Milozzi, Sergey Tsarev, Rolf Brönnimann, Simon C. Boehme, Erfu Wu, Ivan Shorubalko, Maksym V. Kovalenko, Daniele Ielmini

**Affiliations:** ^1^ Department of Chemistry and Applied Biosciences, Institute of Inorganic Chemistry, ETH Zürich, Zürich CH-8093, Switzerland.; ^2^ Empa-Swiss Federal Laboratories for Materials Science and Technology, Dübendorf CH-8600, Switzerland.; ^3^ Dipartimento di Elettronica, Informazione e Bioingegneria, Politecnico di Milano and IU.NET, Piazza L. da Vinci 32, Milano 20133, Italy.

## Abstract

With increasing computing demands, serial processing in von Neumann architectures built with zeroth-order complexity digital circuits is saturating in computational capacity and power, entailing research into alternative paradigms. Brain-inspired systems built with memristors are attractive owing to their large parallelism, low energy consumption, and high error tolerance. However, most demonstrations have thus far only mimicked primitive lower-order biological complexities using devices with first-order dynamics. Memristors with higher-order complexities are predicted to solve problems that would otherwise require increasingly elaborate circuits, but no generic design rules exist. Here, we present second-order dynamics in halide perovskite memristive diodes (memdiodes) that enable Bienenstock-Cooper-Munro learning rules capturing both timing- and rate-based plasticity. A triplet spike timing–dependent plasticity scheme exploiting ion migration, back diffusion, and modulable Schottky barriers establishes general design rules for realizing higher-order memristors. This higher order enables complex binocular orientation selectivity in neural networks exploiting the intrinsic physics of the devices, without the need for complicated circuitry.

## Teaser

Second-order dynamics in halide perovskite memristive diodes that enables Bienenstock-Cooper-Munro learning rules are presented.

## INTRODUCTION

Digital systems based on von Neumann architectures and built with zeroth-order complexity circuits have carried the workload of computing till date. However, with the exponential growth of computing needs, serial processing in such architectures is quickly saturating in terms of both computational capacity and power, entailing research into alternate paradigms (*1*). Because of their large parallelism, low energy consumption, and high error tolerance, brain-inspired neuromorphic systems are attracting considerable interest, especially for tasks such as classifying billions of images and powering speech recognition services (*2*). At the hardware level of the computing stack, the discovery of memristors has fueled approaches based on intrinsic device dynamics to replace complicated digital circuits, paving way for more efficient and simpler in-memory computing architectures (*3*, *4*). However, most demonstrations have thus far centered only around mimicking primitive lower-order biological complexities using devices with first-order dynamics (*5*, *6*). Although theoretical predictions of the benefits of higher-order devices exist, experimental demonstration of memristors with higher-order complexity is far and few (*7*–*9*). Memristors with higher-order complexities are predicted to solve problems that would otherwise require increasingly elaborate circuits (*10*), but no generic design rules exist.

One of the intriguing features of biological neural networks (NNs) is their plasticity, which helps them to learn through experiential change in configuration. The human brain constantly evolves over time, creating new synaptic associations dependent on lifelong learning experiences and knowledge. Reproducing this ability of plasticity to perform in-memory computations in hardware is at the very core of neuromorphic engineering (*11*). Bearing functional resemblance to biological synapses, memristors are at the heart of such in-memory computing technology, and hence, biorealistic realization of synaptic plasticity in memristors is considered a crucial step toward realizing NNs with high accuracy and unsupervised learning capabilities.

### Need for complex learning rules

In this context, selection of a plasticity model plays a vital role in designing neuromorphic systems. The first generation of neuromorphic systems typically implements some form of the pair or doublet spike timing–dependent plasticity (DSTDP) model—a local event-based weight update scheme that maps synaptic weight changes as a function of the timing between the pre- and postsynaptic spikes (*12*–*15*). This simple timing-based model is highly convenient because it allows for low-power operations within a specifically defined domain. However, the positive-feedback process this paired timing–based model adopts, in which strong synapses are further strengthened and weak synapses are further weakened, does not explain several key aspects of biological plasticity (*16*). It destabilizes the useful dynamic range of synaptic weights and fails to address time-variant problems such as online modeling of dynamic processes in visual surveillance. Hence, we need to look beyond simple DSTDP rules to model the next generation of NNs.

Information processing in the brain involves a high connectivity—each neuron is estimated to be connected to up to 10^4^ other neurons via synaptic junctions. Thus, synaptic plasticity can be intuitively considered to be a multifactor phenomenon. In biology, several factors are hypothesized to contribute to the learning process such as the timing between spikes (*17*), rate of pre- and postsynaptic firing (*18*), historical pattern of activity at the synapse (*19*), and global parameters like electrochemical environment, ionic concentration, and temperature (*20*). Despite the impressive progress already demonstrated with memristor-based computing architectures, many of the abovementioned factors are hitherto unaddressed, entailing innovative hardware approaches to emulate the plasticity and connectivity of biological NNs. This calls for a second generation of neuromorphic materials and devices, whose switching physics are capable of adhering to biorealistic plasticity models that capture both timing- and rate-based correlations, and encompass history-dependent activation and global regulatory controls.

In this work, we show second-order dynamics in halide perovskite semiconductors, an archetypal ionic-electronic material. With a compositional space of >10^6^ formulations that can be explored via solution-based simple processing, halide perovskites, as a material technology platform, offer a wide range of design options for memristive and neuromorphic devices. These materials are relevant for a wide range of neuromorphic architectures because they support a rich variety of switching physics, such as electrochemical metallization reactions with reactive electrodes, valence change mechanisms via halide ion migration, spin-dependent charge transport, and multiferroicity (*21*–*23*). Their mixed ionic-electronic conductivity enables comprehensive demonstration of Bienenstock-Cooper-Munro (BCM) learning rules, capturing both timing- and rate-based plasticity effects in a memdiode configuration. Ion migration and back diffusion result in modulable Schottky barriers at the halide perovskite–transport layer interfaces that are exploited by a triplet spike timing–dependent plasticity (TSTDP) scheme. This protocol establishes general design rules for realizing higher-order memristors with similar ionic-electronic materials.

**Fig. 1.**
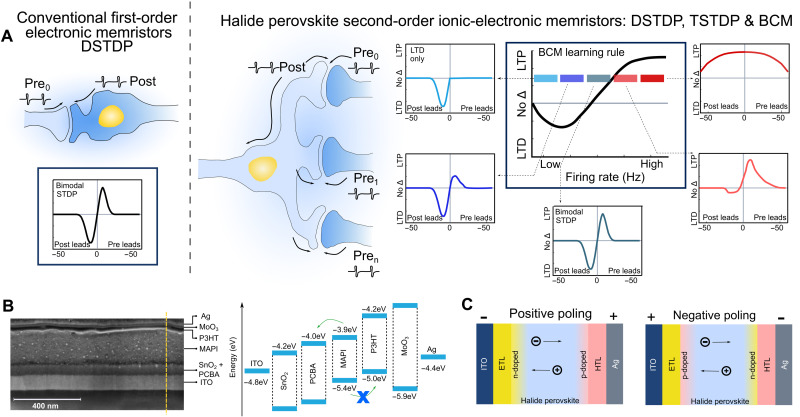
Design of higher-order ionic-electronic memristors. (**A**) Conventional first-order electronic devices are capable of capturing only simple timing-based plasticity rules such as DSTDP (highlighted in the blue box on the left). On the other hand, higher-order memristors can follow a multifactor BCM learning rule (highlighted in the blue box on the right), where both timing and rate of firing are captured for a more robust learning. High firing rates induce LTP because they evoke strong postsynaptic depolarization and calcium signals, low to moderate firing rates induce LTD because they evoke moderate depolarization and calcium signals, and very low firing rates do not induce plasticity. Plasticity depends on the pre-post spike timing for different ranges of firing rate, illustrated by the colored boxes and arrows (*63*). Thus, the net plasticity reflects an interaction between the pre-post spike timing and firing rate. Here, second-order dynamics are observed in halide perovskite memdiodes with the structure ITO/SnO_2_ + PCBA/MAPI/P3HT/MoO_3_/Ag. (**B**) Scanning electron microscopy cross-sectional image of the sample. The built-in potential due to band alignment and the Schottky barrier introduced at the MAPI-P3HT interface allows tunable temporal dynamics, a critical design feature of the second-order halide perovskite memdiode. (**C**) The intrinsic ion/ion vacancy migration in halide perovskites locally dopes the perovskite–transport layer interfaces, enabling finely modulable conductance/weight changes. The back diffusion of ions introduces an additional rate dependency, which we exploit to capture the BCM learning rules.

Going beyond the conventional Hebbian learning rule, the BCM rule is a biorealistic pattern-based plasticity law that captures the effect of both the timing between paired spikes (as in the case of common DSTDP) and the spike train rate, also known as spike rate–dependent plasticity (SRDP), and describes history-dependent synaptic modification (Fig. 1A). In contrast to previous investigations that use SRDP and DSTDP schemes (*24*–*26*), we exploit the TSTDP plasticity model (*27*) to map BCM rules in our memristive diodes, also known as memdiodes. Using a spike train stimulation protocol, we faithfully emulate the high connectivity of biological neurons and demonstrate advanced plasticity features, going beyond simple synaptic learning functions previously shown using single and paired spikes, e.g., excitatory postsynaptic current (EPSC), paired-pulse facilitation (PPF), and DSTDP. The migration and back diffusion of ions in halide perovskites introduce an internal timing factor akin to Ca^2+^ dynamics in biology that, together with a last spike–dominating rule and state-dependent forgetting effects, captures both temporal and rate-based correlations. We successfully demonstrate two main characteristics of the BCM rule, frequency dependence and sliding threshold (*28*), and establish a negative feedback process to regulate synaptic weight updates within a useful dynamic range, thus improving the stability of the NN. Inspired by the BCM rules that explain orientation selectivity in the mammalian visual cortex, we develop simulations of binocular orientation–selective NNs where the mechanism of plasticity involves temporal competition between input patterns instead of spatial competition between synapses as in Hebbian learning. We demonstrate all the features predicted by BCM learning with memristive devices.

## RESULTS

### Design of halide perovskite memdiodes

The halide perovskite memdiodes have the following structure: indium tin oxide (ITO; 83 nm)/tin oxide (SnO_2_, 60 nm) + [6,6]-phenyl-C_61_-butyric acid (PCBA; ~1 nm)/methylammonium lead iodide (CH_3_NH_3_PbI_3_, MAPI; 207 nm)/poly(3-hexylthiophene-2,5-diyl) (P3HT; 19 nm)/molybdenum trioxide (MoO_3_; 11 nm)/silver (Ag; 30 nm) (Fig. 1B). In ABX_3_ halide perovskites [A = CH_3_NH_3_, CH(NH_2_)_2_, Cs, Rb; B = Pb, Sn, Ge; X = I, Br, Cl, F], the soft lattice allows easy diffusion of ions across the octahedral structure, resulting in intimate coupling of ionic transport with electronic transport (of electrons and holes). Ionic transport is the process of hopping between ions’ equilibrium locations in interstitials, defects, or defect hopping (*29*). Hence, halide perovskite is an archetypal mixed ionic-electronic conductor (*30*). A vast body of evidence supporting the idea of ion movement in halide perovskites has been uncovered through careful spectroscopy studies, electrical parameter evaluations, device modeling, and microscopic simulations (*31*–*35*). Although the migration of halide (X) vacancies is the most favored kinetics (*36*, *37*), under external driving forces such as voltage, light, and temperature, all the different types of ions (A, B, and X) will move in the halide perovskite structure (*36*, *38*, *39*).

In our devices, we show second-order switching dynamics and ascribe this to ion drift under the electric field in the perovskite layer (Fig. 1C). Theoretical calculations and experimental observations have substantiated localized p- and n-type doping under the accumulation of negatively charged Pb (*V*_Pb_′) and MA (*V*_MA_′) vacancies and positively charged I (*V*_I_*) vacancies, respectively (*40*, *41*). Upon applying positive bias to Ag, we hypothesize the migration of negatively charged *V*_Pb_′ and *V*_MA_′ toward the hole transport interlayer, locally p-doping the perovskite-P3HT interface. Parallelly, the positively charged *V*_I_* n-dopes the SnO_2_ + PCBA-perovskite interface, forming a p-i-n structure. As a result, the Schottky barriers at these interfaces are modulated, resulting in analog-type resistive switching (*13*). P3HT is chosen specifically to introduce a significant Schottky barrier with MAPI at the hole extraction side as indicated in the band diagram (Fig. 1B). The flavor of resistive switching can be tuned to emulate both short- and long-term plasticity of biological synapses based on the input stimulation (*12*). Upon removing bias, the ion vacancies can relax back to their initial or new metastable states depending on the history of stimulation. This decay caused by the back diffusion of the ions or ion vacancies introduces an additional rate factor, which we exploit for emulating BCM learning rules using a TSTDP scheme as detailed below. Furthermore, reverse biasing flips the p-i-n structure to n-i-p by forcing ions or ion vacancies to drift in the opposite direction. The concept of localized p- and n-doping is further supported by the observation of photoluminescence (PL) quenching at the respective interfaces (note S1 and fig. S1).

### Simple learning rules: Timing-based plasticity

**Fig. 2.**
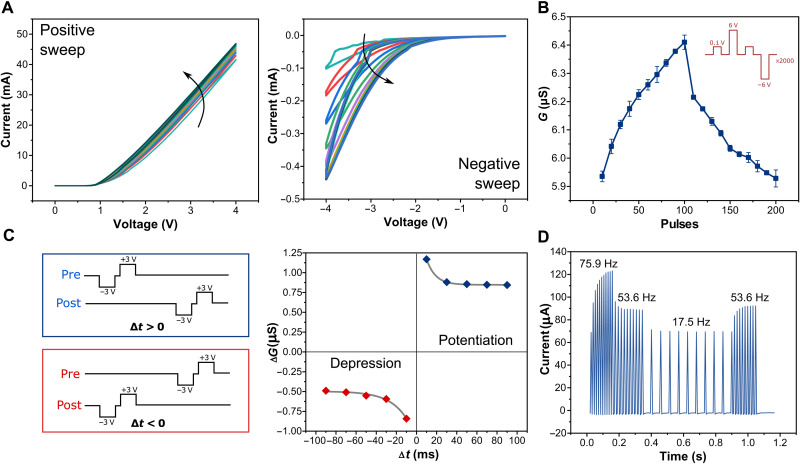
Simple learning rules: Timing-based plasticity. (**A**) *I*-*V* curves of a halide perovskite memdiode under continuous positive (0 to 4 V) and negative (0 to −4 V) sweeps. (**B**) LTP and LTD of synaptic weights/conductance (*G*) caused by repeated stimulation of 100 positive [+6 V, 100 ms] and 100 negative [−6 V, 100 ms] pulses. All states were read 1 s after the stimulation using a reading pulse of +0.1 V, 100 ms. The statistics shown as box plots represent 2000 write-erase operations. (**C**) Emulation of Hebbian DSTDP learning rules with the perovskite memdiodes. Here, the time lag Δ*t* = *t*_post_ − *t*_pre_ between presynaptic and postsynaptic spike times determines LTP and LTD. (**D**) Response of the excitatory postsynaptic currents to a group of spike trains [−1 V, 10 ms] with a frequency sequence (75.9 Hz → 53.6 Hz → 17.5 Hz → 53.6 Hz).

We begin by demonstrating simple timing-based plasticity in our devices. Figure 2A shows *I*-*V* curves of the halide perovskite memdiode, gradually increasing and decreasing in conductivity with positive- and negative-bias sweeps on the Ag electrode. The asymmetric *I*-*V* curves indicate the existence of Schottky barrier at the perovskite–transport layer interfaces. The continuous adjustment of conductance/synaptic weight resembles the nonlinearity of a biological synapse. Analogous to PPF in biology, the devices show short-term synaptic plasticity, which manifests itself as an enhancement in the amplitude of the second of the two rapidly evoked excitatory postsynaptic currents (note S2 and fig. S2, A to C) (*6*). This is critical for a synapse to make correlations between the temporal spike pairs. Upon repeated stimulation, the synaptic weights transit from short- to long-term states, and demonstrate potentiation and depression with good signal-to-noise ratio and low write noise (Fig. 2B). Transient dynamics of the raw currents reveal spontaneous decay to intermediate metastable states, indicating second-order dynamics in the halide perovskite memdiode (note S2 and fig. S3) (*42*). This can be attributed to the drift of ionic vacancies under the external bias and their back diffusion upon removal of the bias under the built-in electric field present in the device, paralleling the influx and extrusion of Ca^2+^ through synaptic cells. This, in turn, results in a permanent change of the Schottky barrier at the MAPI-P3HT interface (Fig. 1C), mimicking the intracellular neural membrane potential. Last, we implement the DSTDP learning rule using nonoverlapping spikes in our devices. Contrary to most implementations using heavily engineered overlapping spikes (*43*), here, the physics of the devices determine the magnitude and polarity of the weight update, mitigating the need for complex peripheral circuitry. Here, the weight modification *G*(Δ*t*) after a spike pairing depends in a characteristic way on the time lag Δ*t* = *t*_post_ − *t*_pre_ between presynaptic and postsynaptic spike times. Around Δ*t* = 0, the DSTDP model assumes a sharp transition from maximal long-term depression (LTD) to maximal long-term potentiation (LTP). For Δ*t* > 0, we observe LTP, and LTD for Δ*t* < 0 (Fig. 2C), following Hebbian rules.

The devices also exhibit history-dependent plasticity at the short-term memory scale—a form of pseudosynaptic adaptation. To demonstrate this, we first apply a series of postsynaptic high-frequency (75.9 Hz) and low-frequency (17.5 Hz) patterns to the device to mimic “experience.” This phase defines the history of the device and sets the value of the experienced conductance *G*_0_. Next, a phase of 53.6-Hz spikes is applied to monitor the device response. As shown in Fig. 2D, it is interesting to note that the same inputs (53.6-Hz spikes) induce contrasting changes in conductance based on the previous experience. When the device has first experienced low-frequency (17.5 Hz) patterns, it exhibits a potentiation behavior to the 53.6-Hz spike inputs. However, an initial experience of high-frequency (75.9 Hz) patterns produces a depression trend to the same 53.6-Hz spike inputs. Systematic studies with different experienced devices reveal a monotonic trend in depression behavior with low-frequency activation and potentiation with high-frequency activation, contradictory to the ideal homeostatic rules seen in biology (note S2 and fig. S4). This behavior deviates from rate-based learning rules in biology because of the lack of (i) long-term changes to the memory (these are short-term changes) (*28*, *44*, *45*), (ii) a multiplicative relationship between presynaptic and postsynaptic neuron activities (these are responses to postsynaptic activities alone), and (iii) a nonmonotonic dependence on spike rate with an enhanced depression effect (EDE) (here, a monotonic trend exists with no EDE region) (*28*). Therefore, a higher adherence to biological learning rules is needed for the device characteristics to enable biorealistic, brain-like cognitive learning.

### Complex BCM learning rules using a TSTDP model: Timing- and rate-based plasticity

**Fig. 3.**
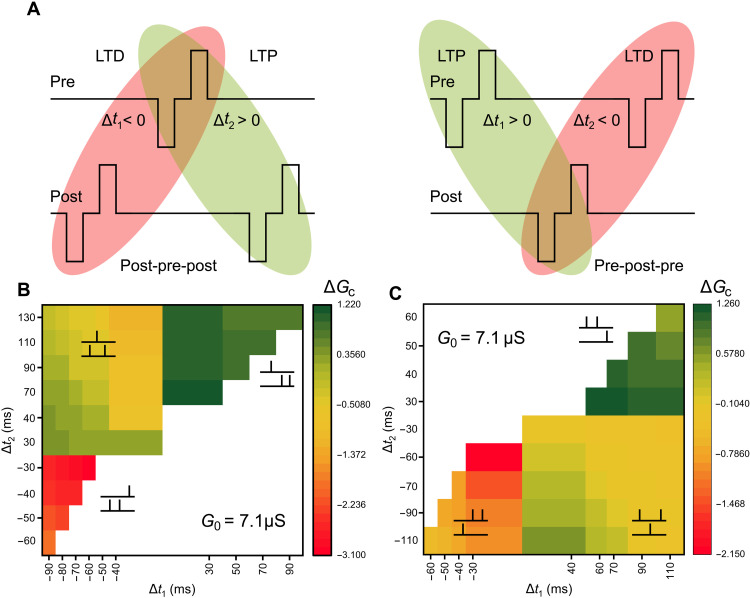
Experimental demonstration of BCM learning rules using TSTDP schemes in halide perovskite memdiodes. (**A**) Schematic of the typical post-pre-post and pre-post-pre triplet inputs applied to the devices. The weights are modified by the timing between each pulse-pair combination (similar to DSTDP) and also the superposition of the LTP and LTD processes (rate dependent). Each pre/postsynaptic spike pattern comprises hundred pulses with an amplitude of ±3 V and a pulse width of 50 ms. For the post-pre-post combination, LTD is activated in the first post-pre pair, with a spike timing of Δ*t*_1_ < 0. This is followed by an LTP process induced by the second pre-post pair, with a spike timing of Δ*t*_2_ > 0. (**B** and **C**) Summary of the experimental TSTDP results. LTP and LTD are induced using different spike sequences and timing intervals, as indicated by the insets. Δ*G* units are in μS.

To address the aforementioned issues, we subject the devices to sequences of specific spike patterns as shown in Fig. 3A and experimentally extract BCM learning rules in our devices based on a TSTDP model. In the TSTDP scheme, each individual spike applied on the memristor has the same shape as for DSTDP but introduces an additional triplet term that interacts with these spikes. Hence, the net spike sequence is assumed to be a combination of two spike pairing events, and the weight change is an integration of the LTP and LTD processes induced by these two events (*27*). However, this is not a direct summation of the two events because the weights are further modified by interaction with the additional triplet term, thus capturing both timing- and rate-based effects in the learning rule. Figure 3A illustrates two typical triplet sequences adopted for analysis—“post-pre-post” and “pre-post-pre.” For the post-pre-post triplet, LTD is induced by the first pairing (“post-pre,” Δ*t*_1_ < 0) and LTP by the second pairing (“pre-post,” Δ*t*_2_ > 0). For the pre-post-pre triplet, the order of LTD and LTP activation is reversed as indicated in the figure. The results of this extensive testing protocol are presented in Fig. 3 (B and C). The other sequence types, namely, “pre-post-post,” “post-post-pre,” “pre-pre-post,” and “post-pre-pre,” are shown as insets. The synaptic weight change (Δ*G*_c_) as a function of the timing intervals (Δ*t*_1_ and Δ*t*_2_) reveals a last spike–dominating TSTDP behavior in our devices.

For the case of pre-post-post, quadrant I of Fig. 3B, LTP is observed for all combinations of Δ*t*_1_ and Δ*t*_2_. Large weight changes are observed when the spike pairs are closely spaced and the triplet interaction is strongest, i.e., for low numerical values of Δ*t*_1_ and Δ*t*_2_. With longer intervals, the weight change decreases as expected. For the case of post-pre-post, quadrant II of Fig. 3B, the weight changes transit from LTD to LTP as Δ*t*_1_ becomes larger or Δ*t*_2_ becomes smaller or a combination of both and vice versa. The net magnitude of weight change depends on a nonlinear integration of the post-pre and pre-post combinations. For the case of post-post-pre, quadrant III of Fig. 3B, LTD is observed for all combinations of Δ*t*_1_ and Δ*t*_2_. Large weight changes are once again observed when the spike pairs are closely spaced and the triplet interaction is strongest, i.e., for low numerical values of Δ*t*_1_ and Δ*t*_2_. Similar logic follows for all quadrants in Fig. 3C. For the case of pre-pre-post, quadrant I of Fig. 3C, LTP is induced by both the spike pair combinations. The weight changes transit from LTD to LTP as Δ*t*_1_ is reduced or Δ*t*_2_ is increased or a combination of both and vice versa for the case of pre-post-pre, quadrant IV of Fig. 3C. Last, LTD is observed for all cases of pre-post-post, quadrant III of Fig. 3C. In all these cases, the device conductance is read out after a delay time of 120 s to ensure stable long-term states. Please refer to note S3 and fig. S5 for a simplified version of Fig. 3 (B and C) and fig. S6 for details of the testing protocol.

The synaptic weight change Δ*G*_c_ shows an additional dependence on the pre-(ρ_x_) and postsynaptic (ρ_y_) spike rates, as shown in note S3 and fig. S7. Here, ρ_x_ is defined as 1/Δ*t*_r_, where Δ*t*_r_ = *t*′_pre_ − *t*_pre_ (time interval between two presynaptic spikes), and ρ_y_ is defined as 1/Δ*t*_o_, where Δ*t*_o_ = *t*′_post_ – *t*_post_ (time interval between two postsynaptic spikes), and they are considered to be equal in this case. The results indicate a directly proportional enhancement of LTP and an inversely proportional enhancement of LTD with increasing spike frequency, consistent with the biological TSTDP rule and contrasting to the DSTDP rule.

### BCM rule as explanation for binocular direction selectivity in mammal visual cortex

**Fig. 4.**
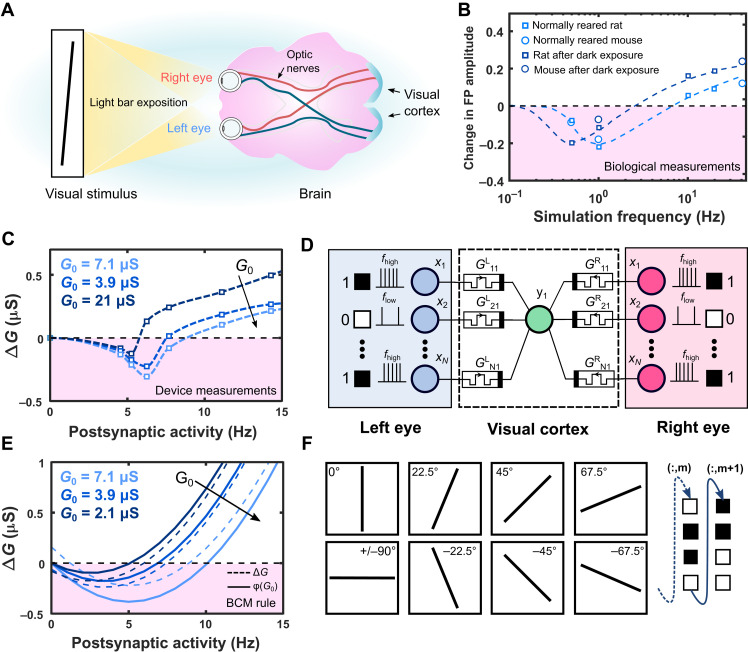
From biological BCM rule to the visual cortex network. (**A**) Binocular vision in mammalian brain: Visual inputs from both eyes propagate through optic nerves reaching visual cortex in the back part of the brain. Here, the neurons show binocular orientation selectivity (*19*). (**B**) Biological measurements of BCM rule on rats and mice with different ambient exposition [data reproduced from (*44*, *47*)]. There are two regions of potentiation and depression depending on the neural activity of the post neuron. Moreover, the threshold between potentiation and depression depends on the history of neural activity. (**C**) Device measurements of halide perovskite memdiodes: The behavior of these devices resembles the synaptic dynamics of biological curves (B). Potentiation and depression regions for each curve can be clearly identified. The separation value between these two regimes is not fixed, but it depends on the history of postsynaptic activity. (**D**) Binocular orientation–selective network: There are two input layers, one for each eye, where the correlated patterns are fed. The first layer is connected to the cortical neuron through synapses that in this case are modeled on the halide perovskite memdiode characteristics. (**E**) BCM model: We choose a parabolic shape for ϕ, indicated with full lines. Dotted lines instead show the weight updating curves for synapses that take into account also the ρ(*w*) term. This simple shape is also the one originally proposed by Bienenstock *et al.* (*19*) and is of common use for BCM applications. The main idea is to properly follow the separation between LTP and LTD as a function of the history of postsynaptic activity. This request is fulfilled by our model based on the device threshold behavior. (**F**) Inputs of the simulated NN mimicking different orientations of visual exposure. The inputs are built as 100 × 100 matrices, which are transformed into a 10K array, fed to the first layer of the NN.

The BCM rule was originally proposed to explain biological measurements showing input selectivity observed in the mammal visual cortex (*19*). In particular, it was observed that cortical cells are binoculars, receiving inputs from both the eyes through optic nerves that, reaching the lateral geniculate nucleus, send the signals to the visual cortex (Fig. 4A). In this specialized brain area, cortical cells are locally selective to specific inputs after exposure to different stimuli such as differently oriented light bars showing orientation selectivity (*28*, *46*). It was observed that the properties of the cortical cells are modified by the visual experience of the animal (*28*), such as in a metaplasticity behavior. In the underlying biological mechanisms, there is a specific dynamics for cortical plasticity. Measurements on biological samples reveal temporal dependence of the synaptic weights (both the sign and magnitude) on the postsynaptic response with a threshold dividing potentiation from depression. As shown in Fig. 4B reported from (*44*, *47*), this threshold is not fixed in time, but it depends on the history of the postsynaptic activity. This results in a temporal competition between input patterns, different from Hebbian-related plasticity mechanisms that involve a spatial competition between synapses (*19*, *28*). For this reason, the BCM rule opens the possibility to explain high-order spatiotemporal neural mechanisms in the visual cortex (*48*) and can enable high-order neuromorphic functions in hardware.

From a mathematical point of view, the general BCM rule describes the synaptic weight change as

$$\dot{w}=\alpha \varphi [y(t)]x(t)-\rho (w)$$ (1)

where *x*(*t*) is the presynaptic activity, α is a fixed learning rate, ϕ is a function of the postsynaptic activity *y*(*t*), and ρ(*w*) is a uniform term to account for metaplasticity. ϕ determines the sign of the variation of synaptic weight for ρ = 0 and in particular

$$\left\{ \begin{array}{rl} \varphi [y(t)] & >0\;\mathrm{if}\;y(t)>\vartheta_{m}(t)\\ \varphi [y(t)] & <0\;\mathrm{if}\;y(t)<\vartheta_{m}(t) \end{array}\right.$$ (2)

where ϑ*_m_* is a threshold with dimensions of activity that, by definition, separate positive variation (potentiation) from negative variation (depression) of the synaptic weights. This value is not fixed, but it depends on the history of postsynaptic activity such that we can write the variation of the weight as

$$\dot{w}=\alpha \varphi (y,\bar{y})x-\rho (w)=\alpha \varphi (y,\vartheta_{m})x-\rho (w)$$ (3)

In particular, ϑ*_m_*(*t*) is a nonlinear function of time-averaged postsynaptic activity $\bar{y}$ that determines the long-term synaptic weight (*19*). As reported before, the ρ(*w*) term is introduced to include the dependence of the dynamics by the value of the weight itself (note S4 and figs. S8 to S10).

Using the TSTDP-based mapping presented in Fig. 3 (B and C) as a guideline, we experimentally demonstrate BCM learning rules in our halide perovskite memdiodes (Fig. 4C). For analysis, we choose the case of post-pre-post triplet with |Δ*t*_1_| = |Δ*t*_2_| (refer to the diagonal of quadrant II of Fig. 3B). The weight changes are monitored for three values (2.1, 3.9, and 7.1 μS) of the experienced conductance *G*_0_ as a function of the postsynaptic spike rate ρ_y_, defined as 1/(|Δ*t*_1_| + |Δ*t*_2_|). The synaptic weight changes Δ*G*_c_ depict a nonmonotonic dependence with the spike rate, transitioning from depression to potentiation with a threshold value ϑ_*m*_. The depression behavior is enhanced at low spike rates for all values of *G*_0_, clearly indicating an EDE region, absent in previous implementations. Moreover, the threshold ϑ_*m*_ appears to be modulable, increasing for strongly experienced systems, i.e., large *G*_0_, and reducing for inactive scenarios (low *G*_0_). This sliding threshold effect faithfully replicates the BCM curve observed in biology. We can clearly identify two regions of the synaptic weight (potentiation and depression) depending on the postsynaptic activity. Moreover, the threshold value that separates the two regions depends on the initial conductance state *G*_0_ of the device, i.e., the history of postsynaptic activity, as described in the previous section.

### Bioinspired second-order NN demonstrates binocular direction selectivity

The BCM learning rule finds application in designing binocular orientation–selective networks. To demonstrate this, we simulate a feedforward neural network (FNN) where synapses are realized with the halide perovskite memdiodes. It is important to note that the positive and negative variations of the synaptic weight and the sliding threshold dependence on the time-averaged output activity are intrinsic to the physics of the devices, rather than a complex circuit deployment. As illustrated in Fig. 4D, we simulate two different input layers, one for the right eye and one for the left eye. These layers are connected through the memdiode synapses to the visual cortex cell. To implement the simulation of the FNN synapses, we extract $\dot{w}$ from the device characteristics, choosing for ϕ(*y*, ϑ*_m_*) the classical form

$$\varphi (y,\vartheta_{m})=y(y-\vartheta_{m})$$ (4)

It should be noted that the parabolic shape is an idealization to capture the essential features with a simple model. This is a common use approximation in neuroscience (*28*). As reported before, the real BCM curves measured in biological cells show a more complex shape that can be simplistically described with a parabolic model (*42*, *48*, *49*). The crucial point for the model is to properly follow the dynamics of the threshold between potentiation and depression. This is well described in our model as reported in Fig. 4E, where variations of the weight and ϕ(*y*, ϑ*_m_*) are reported as a function of *G*_0_. In the simulation, the *G*_0_ value is obtained with a temporal moving average on *G*(*t*) that is compatible with effect of average postsynaptic activity on BCM rule (*50*) and physical updating properties of the perovskite memdiodes. This value determines the moving threshold, i.e., which particular curve we use to update *G*(*t*). Once the specific BCM curve is determined, the variation of the weight is calculated with Eq. 3 and the synaptic weight values are updated (refer to note S4 and figs. S8 to S10 for details).

As shown in Fig. 4F, we select eight different directions as inputs to mimic different orientations of a light bar exposed to the mammal eyes. These inputs are correlated, i.e., both the eyes see the same input. A noise term with randomly distributed pixels is shown with a probability *P* = 0.2 to take into account imperfect correlations and to test the robustness of the system. The input patterns are presented as a 100 × 100 matrix, where the activity is coded with *x*_low_ = 2 Hz for white pixels and *x*_high_ = 20 Hz for black pixels. The matrix is transformed to a 10K array and fed to the first layer of the left and right eye. The inputs are sequentially and randomly shown to the network with the same probability *P* = 0.1 for each pattern, the postsynaptic activity is recorded, and the system is let free to evolve. It should be noted that no winner-take-all or back propagation is present in this network.

**Fig. 5.**
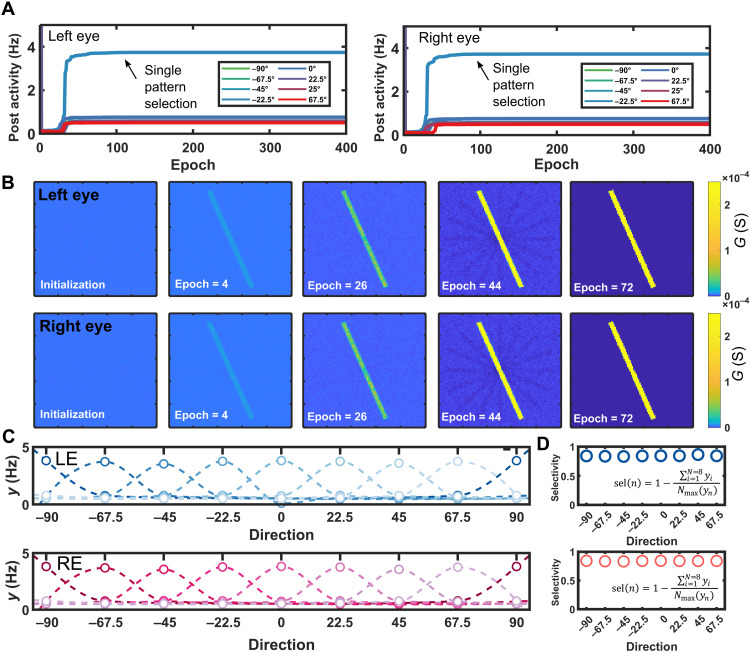
Simulation results for the binocular orientation–selective network. (**A**) Postsynaptic activity due to the exposure of the network to a particular input pattern. Initially, different input patterns show the same postsynaptic activity. After some epochs, the postsynaptic activity becomes higher for a random direction selected by the network, in this case −22.5°. Comparing the left and right eyes, we can see how they select the same direction. Nonselected patterns show a lower response in the activity, whereas slightly higher activity is observed for patterns with smaller rotation with respect to selected pattern, i.e., 0° and −45°. (**B**) Temporal evolution of synaptic weights for the left and right eyes: A random weighted matrix is initialized with μ = 4 μS and σ = 0.1. After consecutive exposure to input patterns, the rise of selectivity can be evidently seen. (**C**) Selectivity of different orientated neurons: We show eight different neurons that select the eight different possible inputs. We can see how the maximum response corresponds to a specific direction. (**D**) Selectivity parameter is nearly constant for all the cortical neurons, showing that the specific input is not relevant in the value of selectivity.

Figure 5A shows how, after some epochs, the postsynaptic activity becomes higher, compared to other patterns, for a specific random chosen pattern selected by the network (−22.5° in this case). Moreover, despite the noise, both the eyes select the same direction, as theoretically predicted by BCM theory. This is due to the presence of correlation between inputs that involves a temporal competition between patterns, which, in the end, is won by the same input (*19*). From a biological perspective, the correlation between signals reaching cortical cells corresponds to a spatial organization in the visual cortex that becomes locally selective to a specific pattern (*51*). As a corollary, the type and the order in which the stimuli reach the cortical cells are crucial to determining the spatial arrangement of selectivity in visual cortex (*52*).

The evolution of the synaptic weights in Fig. 5B illustrates learning of a specific pattern with a small misalignment between the two eyes. In general, the BCM learning mechanism is expected to allow the network to maximize the response of neurons to a particular input after some time of exposure. This is highlighted by Fig. 5C, where the postsynaptic activity for eight different neurons exposed to different temporal arrangement of the inputs is presented. The neurons are responsive such that each one is selective to one of the possible inputs. The postsynaptic activity is observed to be maximum for the selected direction of the neuron (always the same for both eyes comparing the bottom and top plots).

It is also worthwhile to notice that the presentation of “false” patterns results in a nonzero response in Fig. 5A. This residual false-positive response can be attributed to the overlap between the false pattern and the true map of synaptic weights. Even when the bar is orthogonal to the synaptic weights, an overlap will occur at the center of the pattern. This is in good alignment with the BCM rules as well. Orthogonal patterns with respect to the true one will lead to relatively low postsynaptic activity, while patterns with small rotations from the true pattern will show a slightly larger activity (see details in note S4). However, despite this residual response, the selectivity, i.e., the ratio between the response to the true pattern and the average response to the false patterns, is relatively high, as reported in Fig. 5D. The resulting selectivity value is nearly constant and around 0.9, in notable agreement with original results for cortical neurons (*19*). Inspired by the functioning of cortical neurons, these results show the capability for hardware implementation of advanced spatiotemporal pattern recognition networks with binocular topology in a totally unsupervised way.

## DISCUSSION

Performing computing based on the intrinsic device dynamics, where each device replaces complicated digital circuits in a functional sense, is a potential strategy to enable adaptive complex computing (*53*, *54*). Second-order memristors such as the ones presented in this work enable us to capture both timing- and rate-based learning rules using the devices’ intrinsic physics (*7*). In comparison to digital circuit implementations of higher-order synapses and first-order memristors (*55*), these devices portray advantages in area and circuit complexity.

The need for second-order memristor comes from the complexity of implementing synaptic learning rules with first-order memristors. In the latter devices, the implementation of plasticity rules, such as spike time dependent plasticity (STDP), requires to encode the timing information in the shape of programming pulse. The memristor is used as a simple programmable memory in which the overlapping of spikes results in the right shape of amplitude and duration to encode the timing between presynaptic and postsynaptic neurons. These mechanisms are necessary because there are no other ways to encoding timing information in a first-order memristor. Instead, in second-order memristors, because of a second internal state variable, the activity of synapse controls the plasticity rather than the amplitude or pulse duration. The history of activity of the memristor is stored in the device itself and influences the future behavior (*7*).

In our work, the halide perovskite memdiodes show second-order characteristics encoding timing and rate of spikes, because of their mixed ionic-electronic conduction. The possibility to encode this information in the activity of the synapses instead of a particular shape and/or duration of the pulses permits the use of these devices as second-order elements instead of a simple memory element for high-complexity neuromorphic computing. These devices act as a new building block to implement algorithms and systems without the need for complicated timing circuitry and unaffordable system complexity that first-order elements and digital implementations require. High complexity in this context refers to all neuromorphic computing systems in which a simple first-order memory element is not sufficient to implement the desired learning rule or algorithm, such as the BCM learning rule.

**Fig. 6.**
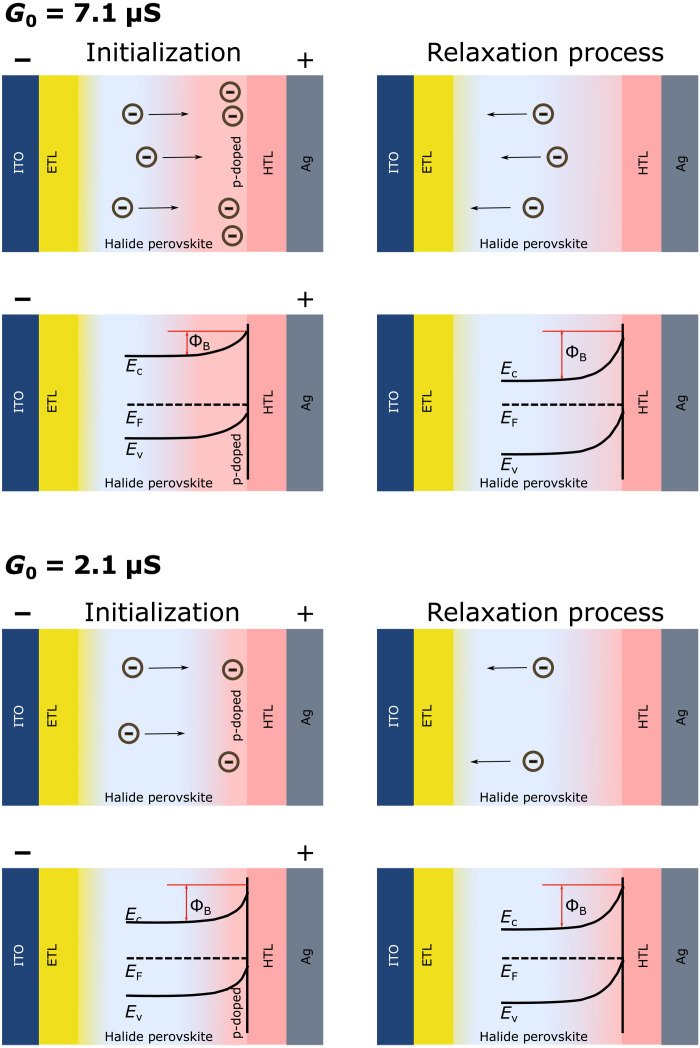
Mechanistic illustration of frequency dependence and sliding threshold of BCM learning rules with halide perovskite memdiodes. Schematic diagram of the memristive mechanism showing the accumulation of ion vacancies, dynamic change in energy band alignment of the MAPI-P3HT Schottky interface, and ion vacancy relaxation at different memristive states.

Because of the specific physical properties of our devices, we successfully demonstrate the two main characteristics of the BCM rule, namely, the frequency dependence and the sliding threshold. The weight update trace reveals multiplicative correlations between presynaptic and postsynaptic activities and a nonmonotonic behavior in the depression region (EDE)—features that previous investigations (*24*–*26*) with SRDP and DSTDP schemes fail to address. In comparison to filamentary memristors, these devices have a larger dynamic range due to the rate-dependent negative-feedback process and the EDE region. The richer dynamics can be attributed to the back diffusion of ionic vacancies that introduce an additional modulatory mechanism along with the inbuilt electronic Schottky barrier (due to band alignment) and stimulation history. As mentioned before, P3HT is chosen specifically to introduce a significant Schottky barrier with MAPI at the hole extraction side (Fig. 1B), and thus, we focus on this part of the device. For analysis, we compare the initial states of a (i) low-experienced conductance state *G*_0_ = 2.1 μS, (ii) medium-experienced conductance state *G*_0_ = 3.9 μS, and (iii) high-experienced conductance state *G*_0_ = 7.1 μS. The two extreme states are shown in Fig. 6. Here, the Schottky barriers arising from the ionic vacancy accumulation are schematically represented for qualitative understanding. As shown, the high-experienced conductance state *G*_0_ = 7.1 μS has a smaller Schottky barrier when compared to the low-experienced (*G*_0_ = 2.1 μS) and medium-experienced (*G*_0_ = 3.9 μS) conductance state due to accumulation of large number of negatively charged *V*_Pb_′ and *V*_MA_′ during the initialization process. Consequently, upon bias removal, more metastable ionic vacancies exist at the MAPI-P3HT interface for back diffusion in the case of *G*_0_ = 7.1 μS, resulting in larger relative changes in the Schottky barrier, and enhanced forgetting and depression effects.

In comparison to the recent demonstration with second-order oxide memristor (*42*) and two-dimensional (2D) heterostructure memtransistor (*56*), the mixed ionic-electronic conduction of halide perovskites offers a simpler processing route, device architecture, and higher yield approach to implement homeostatic regulatory mechanisms at the individual device level, thus establishing a universal design strategy. While other devices require preprogramming to a high conductance state to enable EDE, our device design allows EDE control via band structure and interface engineering and requires no preprogramming step, resulting in power saving. The above observations are expected to provide inspiration for similar ionic-electronic materials systems, such as lithium-intercalated battery-like synapses (*57*) and proton-doped organic electrochemical transistor–based synapses (*58*, *59*).

These device properties enable the implementation of new learning mechanisms exploiting temporal competition between inputs in contrast to classical Hebbian learning where spatial competition between synapses is captured. Further studies, however, are necessary to investigate different materials with second-order dynamics that can add different physical time constants to cover a large spectrum of temporal processing capability. A large set of second-order devices is required to cover different applications with different specifications of operative frequencies and timing. Moreover, the presence of two state variables in these devices requires a deeper understanding of the underlying physics and suitable models to achieve proper optimization. On this point, it is important to note that the classical BCM model is parabolic, while experiments show a more complex functional shape. However, the perfect fitting of experimental curve is detrimental, increasing the complexity of the model without adding any critical features. The crucial point of this rule is to follow the dynamics of the variation of the weight rather than the absolute value of the variation of the weight (*19*, *28*, *50*). The latter would just result in a small change in the convergence speed, while the dynamics that we properly reproduce determines the properties and stability of the system.

To conclude, simulations of binocular orientation–selective networks (*60*) mimicking visual cortex cells demonstrate an example of the relevance of halide perovskite memdiodes in the context of high-complexity computing: The timing/frequency processing properties of these devices enabled the development of a totally unsupervised system that implements a temporal-competition processing between input patterns, which can also be useful in many other general applications (*25*, *61*). This concept will enable a new generation of NNs with higher-order spatiotemporal functions that are useful to capture time-variance features in dynamic environments (*62*). Natural candidates that could benefit from that are video and audio processing systems, which, with these properties, become more similar to the biological learning mechanisms seen in mammalian brains. Furthermore, self-supervised learning for edge computing and efficient spatiotemporal recognition systems will also benefit from these devices, thus introducing an important new building block, significantly advancing beyond state-of-the-art demonstrations.

## MATERIALS AND METHODS

### Perovskite ink preparation

A total of 175 mg of methylammonium iodide (MAI) (Greatcell Solar) and 507 mg of PbI_2_ (Thermo Fisher Scientific; 99.9%) were dissolved in 900 μl of *N*,*N*′-dimethylformamide and 100 μl of dimethyl sulfoxide under nitrogen atmosphere. The ink was heated at 80°C for 1 hour to aid the dissolution of powders.

### Device fabrication

Glass/ITO substrates (Zhuhai Kaivo, 18 ohm/sq.) were sequentially cleaned with soap, water, acetone, and isopropanol. Then, the ITO slides were ultraviolet-ozone–treated for 15 min and immediately coated with the tin dioxide (SnO_2_) layer. The SnO_2_ layer was obtained by spin coating 10% aqueous SnO_2_ suspension (Alfa Aesar) at 4000 rpm for 40 s, followed by annealing at 100°C for 10 min and at 165°C for 15 min in air. All subsequent steps were performed under inert atmosphere inside a nitrogen glove box. The solution of PCBA (concentrations ranging from 0.1 mg/ml in toluene) was spin-coated at 3000 rpm for 30 s and annealed at 100°C for 10 min. The MAPbI_3_ ink (60 μl) was spin-coated at 4000 rpm and quenched with 400 μl of toluene dropped 10 s after the start of spin coating. The deposited films were annealed for 10 min at 100°C on a hot plate. For P3HT deposition, a solution (15 mg/ml) of P3HT (Lumtech) in toluene was deposited at 4000 rpm for 30 s. Molybdenum oxide (15 nm)/Ag (100 nm) electrodes were evaporated through a shadow mask. The device active area was 0.16 cm^2^ as defined by a shadow mask.

### PL measurements

PL experiments were performed via a FluoTime300 setup (Picoquant), using pulsed excitation at 531.5 nm (80 MHz repetition rate), with illumination and detection from the ITO side, and a detection bandwidth of 3 nm. The PL intensity quenching data were obtained at the PL peak (770 nm). For the time-resolved measurements, the repetition rate was reduced to 20 MHz, to ensure a full decay of the PL signal before arrival of the next excitation pulse, and the detection bandwidth was increased to 27 nm, to accommodate potential spectral shifts of the PL peak. Electrical bias was applied via a voltage source for the in operando studies.

## Supplementary Materials

### This PDF file includes:

Notes S1 to S4 Figs. S1 to S10
