# Supplementary material for: Ionic-electronic halide perovskite memdiodes enabling neuromorphic computing with a second-order complexity
Source: Sci Adv. 2022 Dec 23;8(51):eade0072. doi: 10.1126/sciadv.ade0072 (PMC9788778; doi:10.1126/sciadv.ade0072)
Supplement: Supplementary file 1 — Notes S1 to S4 Figs. S1 to S10 [file sciadv.ade0072_sm.pdf]

Science Advances
AAAS

Supplementary Materials for

Ionic-electronic halide perovskite memdiodes enabling neuromorphic computing with a second-order complexity

Rohit Abraham John *et al.*

Corresponding author: Rohit Abraham John, rohitab.john@gmail.com, rohjohn@ethz.ch; Maksym V. Kovalenko, mvkovalenko@ethz.ch; Daniele Ielmini, daniele.ielmini@polimi.it

Sci. Adv. **8**, eade0072 (2022)
DOI: 10.1126/sciadv.ade0072

This PDF file includes:

Notes S1 to S4
Figs. S1 to S10

Supplementary Note 1: Photoluminescence (PL) Measurements

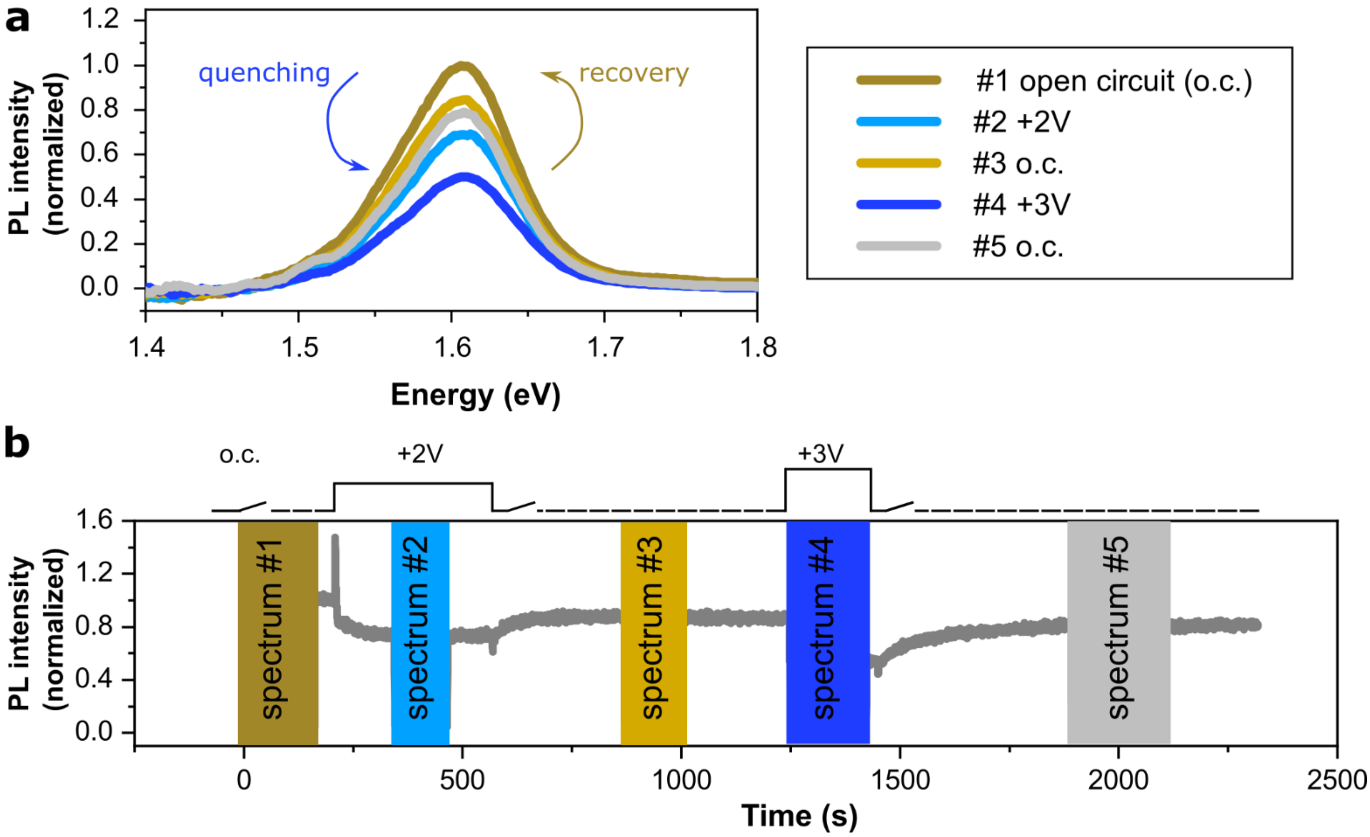

Supplementary Fig. 1. Effect of electrical bias on PL in a halide perovskite memdiode. a PL spectra during selected time points in the trace shown in **b**, illustrating PL quenching upon application of forward bias, i.e. +2V and +3V, and recovery upon return to open circuit. **b** Time trace of the PL peak intensity. Several cycles of +2V, +3V and open circuit are applied during testing. Only 1 representative cycle is shown here. In **a** and **b**, the PL intensity is normalized to the PL peak intensity of the pristine device.

Supplementary Fig. 1a displays experimental evidence consistent with the ion migration theory in halide perovskites. Our device shows reversible quenching and recovery of PL upon cycling between open-circuit condition and forward bias. While the shape of the PL spectrum is bias-independent, application of +3V quenches the PL to about half its intensity under the open circuit (o.c.) condition. Supplementary Fig. 1b reports the reversibility of this process upon cycling between forward bias and open circuit. The PL quenching is reversible and is about twice stronger at +3V than at +2V. We attribute the reduced PL intensity under bias to ion-migration-induced alterations of the Schottky barriers at the contacts, in alignment with literature*(39)*.

Supplementary Note 2: Simple Timing-based Plasticity

Supplementary Fig. 2a shows the current response of the memristive device under a paired-pulse stimulation [-1V, 10ms]. The postsynaptic spike triggers an abrupt increase in current followed by a decay to the initial state within ms, similar to excitatory postsynaptic currents (EPSCs) of a biological synapse. The Ca^{2+} dynamics in a biological system allow for correlation between paired spikes, where the residual Ca^{2+} caused by the first spike boosts the overall Ca^{2+} concentration generated by the second spike, resulting in PPF. In our devices, when the second stimulation comes before the first EPSC disappears completely, the back diffusion of negatively-charged V_{Pb}' and V_{MA}' is effectively suppressed, resulting in larger accumulation of V_{Pb}' and V_{MA}' at the MAPI-P3HT interface, which leads to a larger conductance change. When the intervals are small ($\leq$50ms), the peak value of EPSC induced by the second spike is clearly higher than that of the first spike, resulting in large PPF indices. Longer intervals gradually reduces the PPF indices as shown in Supplementary Fig. 2b. Utilizing spike trains instead of spike pairs, the PPF effect can be extended to demonstrate spike rate dependent plasticity (SRDP) at the short-term memory scale (Supplementary Fig. 2c). Spikes with a higher rate (i.e., a shorter interval) result in clear accumulation of the EPSC amplitude, similar to biological synapses.

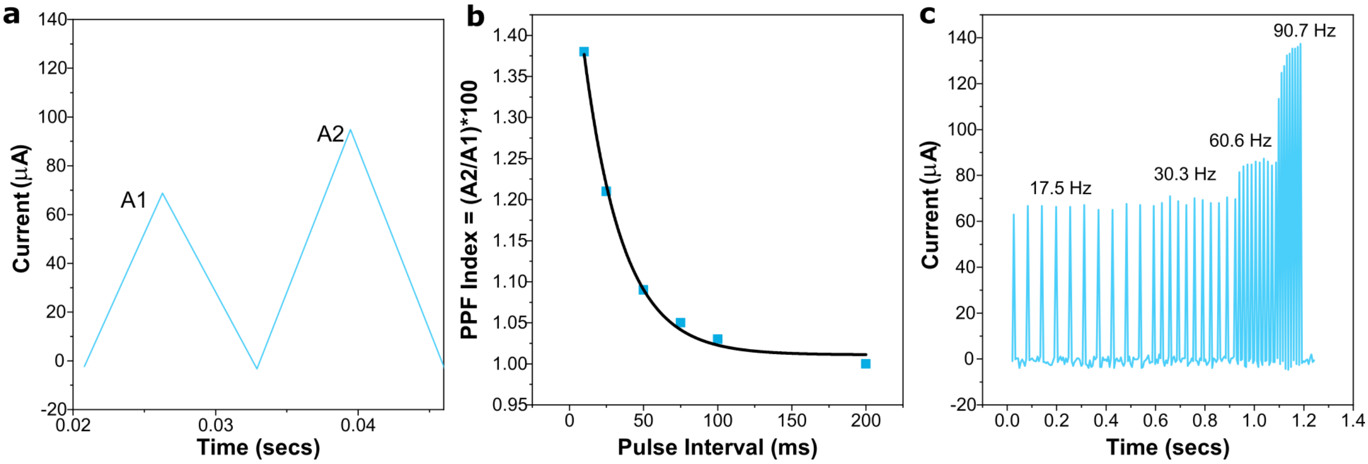

Supplementary Fig. 2. Short-term Plasticity. a A pair of postsynaptic spikes (amplitude = -1V, pulse width = 10ms, interval = 10ms) triggers a pair of EPSCs, with amplitude of the 2nd (A2) higher than the 1st (A1). **b** PPF index as a function of the inter-spike interval. **c** Synaptic weight changes to a group of postsynaptic spike trains (-1V) with various frequencies.

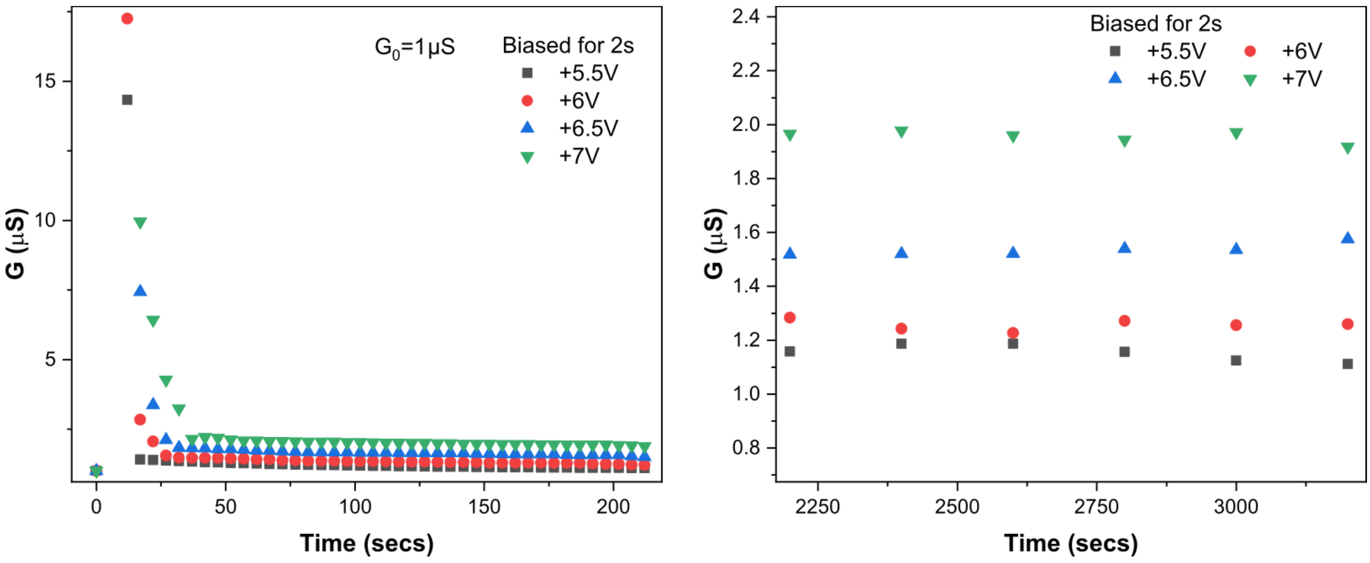

Supplementary Fig. 3. Long-term Plasticity. Transient I-t curves as a function of the input stimulation. The left side shows the decay dynamics and right side shows the retention of the programmed states.

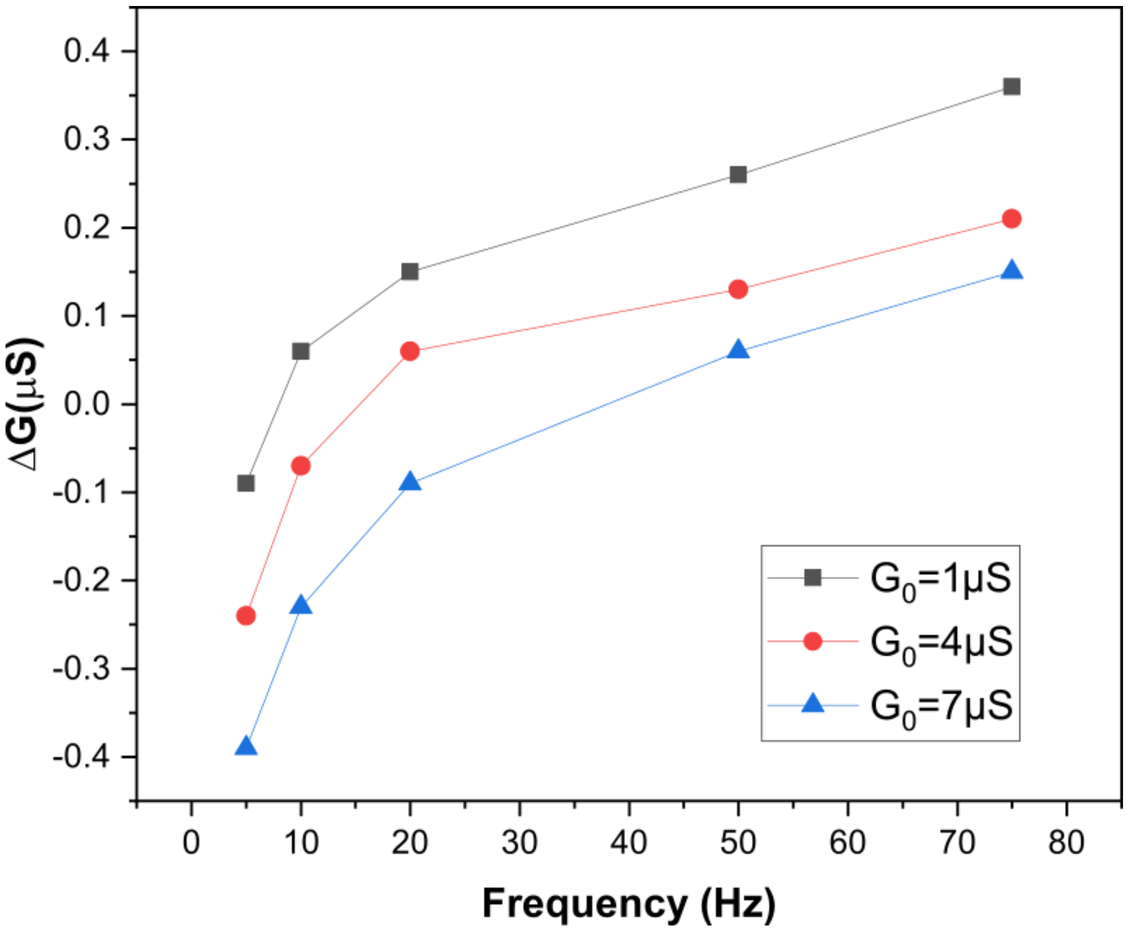

Supplementary Fig. 4. Short-term synaptic adaptation as a function of various learning experiences. Sliding threshold effect of the history-dependent short-term synaptic adaptation function with different G_0. The devices' experience was first initialized to different G_0 as indicated. The devices were then subject to 70Hz stimulation followed by 30Hz. ΔG is calculated as $G_{peak} - G_0$, where G_{peak} is the peak of temporary conductance.

Supplementary Note 3: Timing and Rate-Dependent Plasticity

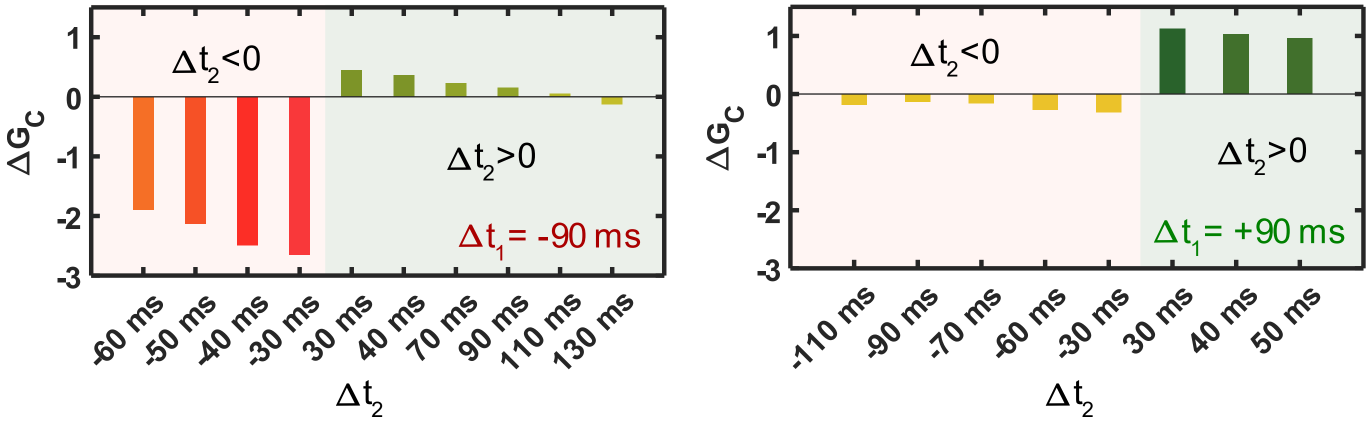

Supplementary Fig. 5. TSTDP with asymmetrical spike timing. Simplified representation of Figs 3b-c (main text). ΔG units are in μS.

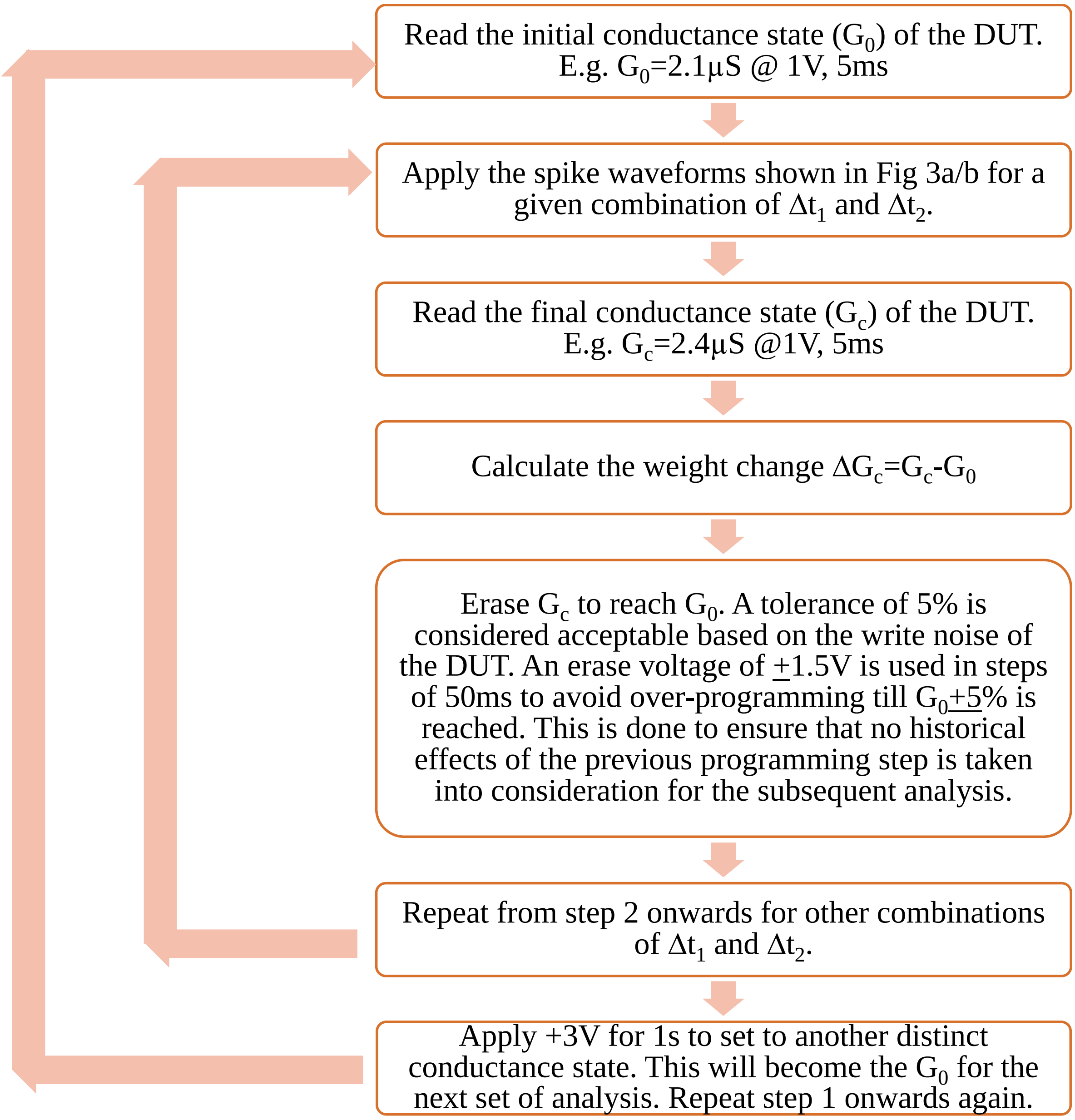

Supplementary Fig. 6. TSTDP testing protocol.

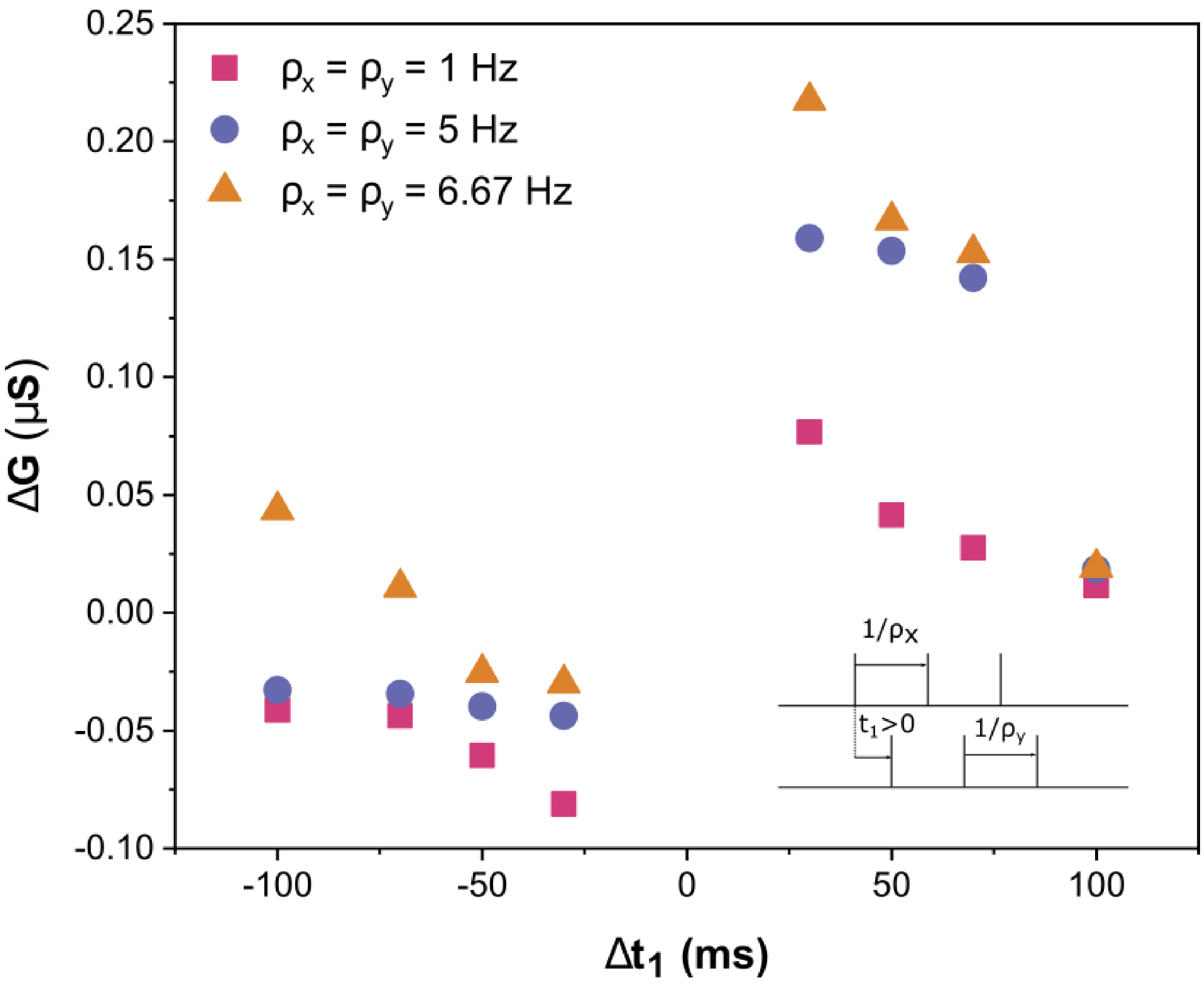

Supplementary Fig. 7. History-dependent long-term synaptic adaptation as a function of various learning experiences. The dependence of ΔG on the presynaptic and postsynaptic spike rates ρ_x and ρ_y respectively.

Supplementary Note 4: Modelling and Simulations

BCM rule and parameters

The BCM rule is generally expressed as:

$$\dot{w} = \alpha\varphi\big(y(t)\big)x(t) - \rho(w)$$

where the equation describes the variation of synaptic weight depending on presynaptic and postsynaptic activity and the value of the weight itself. In particular, the function $\varphi(y)$ is classically implemented with a parabolic shape, where the abscissa-intersection depends on the history of postsynaptic activity. This point divides potentiation from depression, and is not fixed i.e. $\vartheta_m = \vartheta_m(t)$. We use a parabolic shape as well because it is a simple function that describes the important features of BCM rule. In fact, also from a

biological point of view, the shape of the experimental BCM curves are not parabolas. This results in the formulation:

$$\varphi(y, \bar{y}) = y(y - \vartheta_m(\bar{y}))$$

Experimentally, we find that, to fit the moving threshold with the previous relationship, the expression for ρ and ϑ_m are:

$$\vartheta_m = \eta G_0 + 3$$
$$\rho(w) = a{G_0}^2 + bG_0 + c$$

where we do not need to explicitly define the dependence of the threshold on averaged postsynaptic activity because the device itself does this operation. In fact, G_0 value depends on the history of postsynaptic activity and we use this value to determine ϑ_m, coming up with a better physical description in the model with respect to defining an analytical formula that needs more parameters. The overall roots ϑ_A of parabola, taking into account the uniform term becomes:

$$\vartheta_A = \frac{\vartheta_M}{2} \pm \frac{\sqrt{(\alpha x \vartheta_M)^2 - 4\alpha x \rho(w)}}{2\alpha x}$$

that falls into $\vartheta_A = [0, \vartheta_m]$ without the uniform term. The required $\rho(w)$ is in particular to have just real roots of the parabola:

$$\rho(w) < 0.25\alpha x {\vartheta_M}^2$$

which in our case is always true as reported in Supplementary Fig. 7.

Simulations of the network are run on MATLAB R2021a. The synaptic weight updates are done as G(t+1)=G(t)+$\tau\dot{w}$. We include two boundaries for G(t): G_{min}=0S as the lower boundary and G_{max}=250µS as the upper boundary. G_0 value is calculated with a temporal moving average on 6 time points to follow STP of experimental results.

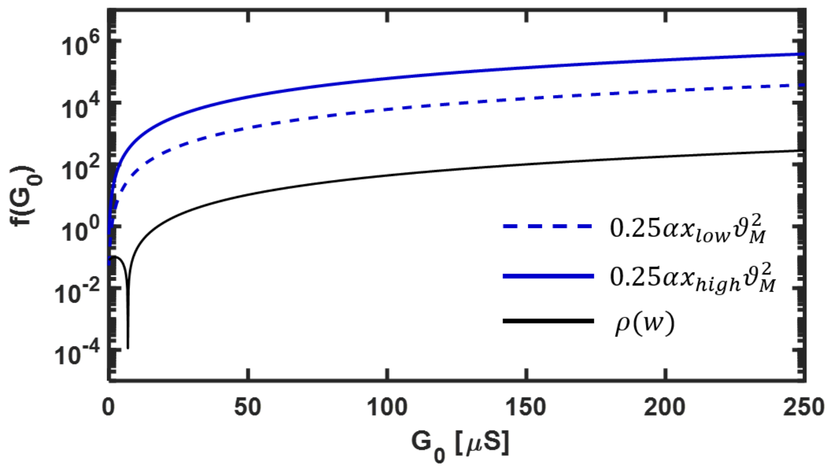

Supplementary Fig. 8. Dependence of Δ elements to find roots ϑ_A. Parameters extracted from the device behavior ensure that real solutions are always obtained for ϑ_A, both for high and low presynaptic activity.

The postsynaptic activity is calculated as:

$$y_j = \sum_{i=1}^{N} x_i G_{i,j}$$

At t=0, the synaptic weights are initialized to random normal distributed values with mean=4μS and sigma=0.1μS. All the functioning on SRDP is intrinsically implemented in the properties of device as schematically reported in Supplementary Fig. 9.

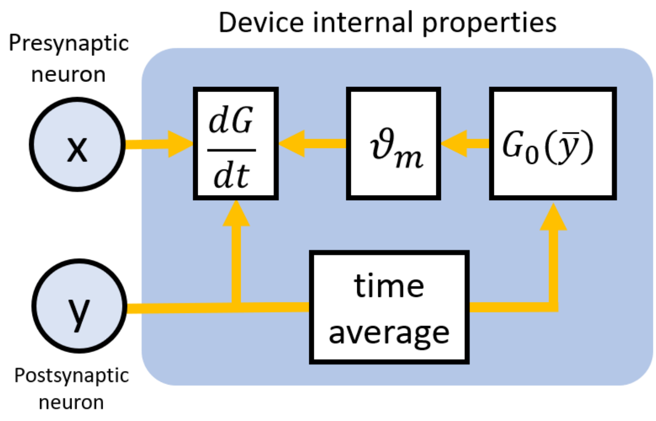

Parameter	Value
η	10^6 [Hz/S]
a	$4.61\text{x}10^9$ [1/S]
b	$-1.96\text{x}10^4$
c	-0.08 [S]
τ	10^{-7}
α	0.003

Supplementary Fig. 9. Block diagram of the evolution of conductance in halide perovskite memdiodes and its parameters. The two terminals of the synaptic device experience the activity of presynaptic and postsynaptic neurons. As described by BCM rule, the magnitude of dG/dt is determined by post and presynaptic activity, such as in classical Hebbian plasticity. Moreover, the sign of dG/dt is determined by a time average intrinsically present in the device, that thanks to the STP, describes the history of postsynaptic activity. The table shows the parameters used for the simulations.

Selectivity definition of BCM rule

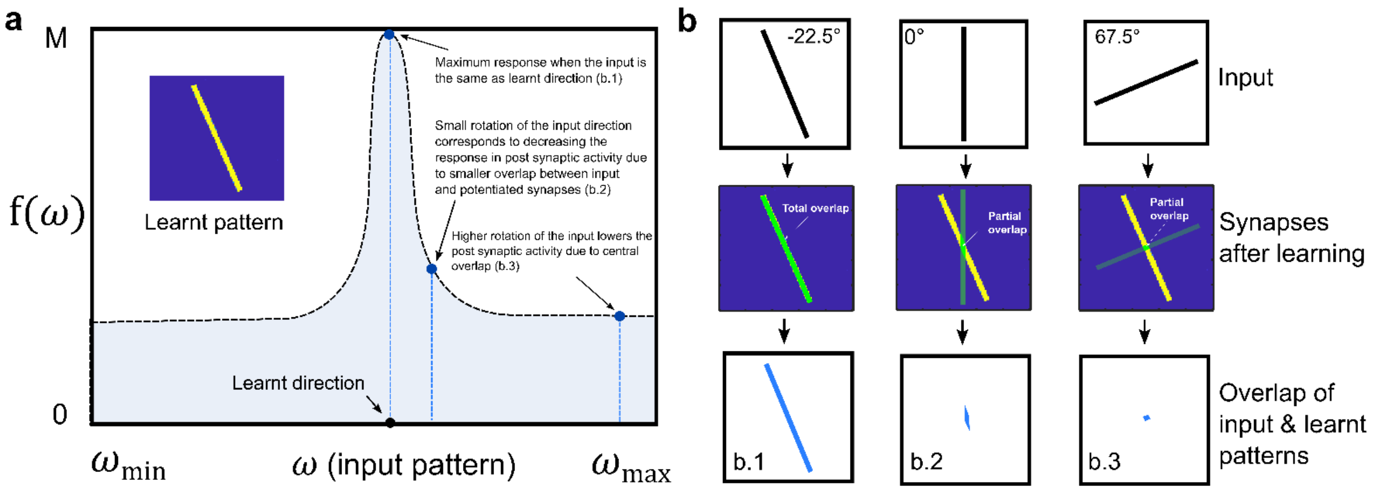

Supplementary Fig. 10. Selectivity definition of BCM rule. a Theoretical selectivity plot for BCM rule: On the x-axis is reported the input vector space, while the function f(ω) (y-axis) is the response i.e. the postsynaptic activity. The function shows a maximum value M for a specific input pattern, while other possible inputs show a lower response. From a geometrical point of view, selectivity is defined as the ratio between light area of the plot and total area of the box plot. **b Explanation for bell-shaped output response:** When the input is equal to the learnt direction, the overlap between potentiated synapses and input corresponds to the maximum postsynaptic activity (b.1). Upon rotating the input, the overlap between potentiated synapses and input direction decreases (b.2), reducing the postsynaptic activity. The limit is for the orthogonal input direction with respect to the learnt pattern, which corresponds to the minimum overlap between potentiated synapses and input direction, and minimum postsynaptic activity (b.3).

The selectivity of the nth pattern is calculated as originally proposed by Bienenstock et al.*(19)*:

$$sel(n) = 1 - \frac{1}{\max(y_n)\,(\omega_{min} - \omega_{max})} \int_{\omega_{min}}^{\omega_{max}} f(\omega)d\omega$$

In our case, due to the finite set of possible directions, this becomes:

$$sel(n) = 1 - \frac{\sum_i^{N=8} y_i}{Nmax(y_n)}$$

where N is the number of possible input patterns.
